# Tracing the Path between Mushrooms and Alzheimer’s Disease—A Literature Review

**DOI:** 10.3390/molecules28145614

**Published:** 2023-07-24

**Authors:** Ana Margarida Silva, Marco Preto, Clara Grosso, Mónica Vieira, Cristina Delerue-Matos, Vitor Vasconcelos, Mariana Reis, Lillian Barros, Rosário Martins

**Affiliations:** 1 Ciências Químicas e das Biomoléculas, Escola Superior de Saúde, Instituto Politécnico do Porto, 4200-072 Porto, Portugal; agl@ess.ipp.pt (A.M.S.); mav@ess.ipp.pt (M.V.); 2 CIIMAR/CIMAR—Centro Interdisciplinar de Investigação Marinha e Ambiental, Universidade do Porto, 4450-208 Matosinhos, Portugal; mpreto@ciimar.up.pt (M.P.); vmvascon@fc.up.pt (V.V.); mreis@ciimar.up.pt (M.R.); 3 REQUIMTE/LAQV, Instituto Superior de Engenharia do Porto, Instituto Politécnico do Porto, 4249-015 Porto, Portugal; claragrosso@graq.isep.ipp.pt (C.G.); cmm@isep.ipp.pt (C.D.-M.); 4 TBIO—Centro de Investigação em Saúde Translacional e Biotecnologia Médica, Escola Superior de Saúde, Instituto Politécnico do Porto, 4200-072 Porto, Portugal; 5 Departamento de Biologia, Faculdade de Ciências, Universidade do Porto, 4169-007 Porto, Portugal; 6 CIMO, Centro de Investigação de Montanha, Instituto Politécnico de Bragança, 5300-253 Bragança, Portugal; lillian@ipb.pt; 7 Laboratório Associado para a Sustentabilidade e Tecnologia em Regiões de Montanha (SusTEC), Instituto Politécnico de Bragança, 5300-253 Bragança, Portugal; 8 CISA, Centro de Investigação em Saúde e Ambiente, Escola Superior de Saúde, Instituto Politécnico do Porto, 4200-072 Porto, Portugal

**Keywords:** mushrooms, Alzheimer’s disease, natural products, therapy

## Abstract

Alzheimer’s disease (AD) is well-known among neurodegenerative diseases for the decline of cognitive functions, making overall daily tasks difficult or impossible. The disease prevails as the most common form of dementia and remains without a well-defined etiology. Being considered a disease of multifactorial origin, current targeted treatments have only managed to reduce or control symptoms, and to date, only two drugs are close to being able to halt its progression. For decades, natural compounds produced by living organisms have been at the forefront of research for new therapies. Mushrooms, which are well-known for their nutritional and medicinal properties, have also been studied for their potential use in the treatment of AD. Natural products derived from mushrooms have shown to be beneficial in several AD-related mechanisms, including the inhibition of acetylcholinesterase (AChE) and β-secretase (BACE 1); the prevention of amyloid beta (Aβ) aggregation and neurotoxicity; and the prevention of Tau expression and aggregation, as well as antioxidant and anti-inflammatory potential. Several studies in the literature relate mushrooms to neurodegenerative diseases. However, to the best of our knowledge, there is no publication that summarizes only AD data. In this context, this review aims to link the therapeutic potential of mushrooms to AD by compiling the anti-AD potential of different mushroom extracts or isolated compounds, targeting known AD-related mechanisms.

## 1. Introduction

Neurodegeneration comprises a series of illnesses that cause the gradual impairment of cognitive and motor abilities. Alzheimer’s disease (AD) is a neurodegenerative disease and the most common form of dementia (60–80% of worldwide dementia cases) [1]. Statistics show that in 2018 more than 8 million people over the age of 30 suffered from AD in Europe (the number is expected to double by 2050), and in the USA, data from 2023 point to nearly 6.5 million persons over 65 years to be affected with AD (incidence of 1 in more than 8 million people over the age of 30 suffered from AD in Europe (the number is expected to double by 2050), and in the USA, data from 2023 point to nearly 6.5 million persons over 65 years to be affected with AD (incidence of 1 in 9) [2,3] Linked to the incidence rate and lack of effective treatment, the high degree of disability and dependency of patients burden personal, medical, and social systems with excessive social and economic costs, which are expected to reach USD 2 trillion by 2030 on a global basis and EUR 330 billion by 2050 in Europe alone [4]. 

AD affects an individual’s ability to perform normal, daily activities, causing dependence, disability, and ultimately, death. It is thought that the disease can be present several years before manifesting any symptoms and, consequently, AD patients will only experience symptoms after a certain level of modification of the brain structure and activity [5]. At a macroscopic level, structural modifications include the age-dependent atrophy of the hippocampus and cerebral cortex, due to neuron degeneration. At a microscopic level, AD hallmarks include the formation of beta-amyloid (Aβ) plaques, due to the aggregation and deposition of Aβ peptide, and neurofibrillary tangles generated by the accumulation of the hyperphosphorylated Tau protein [6,7].

Despite being initially documented more than a century ago, AD lacks an established theory explaining its etiology, progression, and pathogenesis, as well as a treatment that is able to effectively halt disease progression or treat the cause. Nevertheless, some hypotheses have been proposed to explain the origin of AD, namely, the amyloid cascade hypothesis, the cholinergic hypothesis, the tau hypothesis, and the glutaminergic hypothesis [8]. In the first of these hypotheses, the Aβ peptide is produced in excess as a result of the β-secretase (BACE1) cleavage of the Aβ precursor protein (APP). Excessive Aβ undergoes a series of self-assembly steps, resulting in insoluble Aβ aggregates that are deposited extracellularly near the synapses. According to this hypothesis, Aβ aggregates trigger a cascade of events, leading to synaptic dysfunction and neuronal death, manifested in memory loss, and impaired cognitive functions [9]. In the cholinergic hypothesis, the involvement of the acetylcholinesterase enzyme (AChE) is a key factor. Cholinergic neurons in the central nervous system are involved in cognitive processes, such as memory and learning. The stimulation of receptors is caused by acetylcholine (ACh). One of the potential therapeutic strategies to AD is to increase the ACh levels in the synaptic clefts by inhibiting the biological activity of AChE [9,10]. The tau hypothesis relies on the hyperphosphorylation of the microtubule-associated protein tau. When hyperphosphorylated, tau is prone to aggregation and is responsible for neurofibrillary tangle formation. Its intracellular accumulation is associated with reduced neuronal function, impaired synaptic function, and neurotoxicity [6,11]. The glutaminergic hypothesis suggests the inappropriate stimulation of the N-methyl-D-aspartate receptors (NMDARs) as causing the deterioration of synaptic function. NMDARs are involved in synaptic transmission and plasticity. The hyperexcitation of these receptors causes excitotoxicity, which is mediated by increased Ca^2+^, resulting in synaptic dysfunction and neuronal death [12]. Moreover, epidemiological and translational research suggest that AD might also be promoted by neuroinflammation, systemic inflammation, and oxidative stress. Chronic inflammation has been observed as an early event in AD development [13]. In this case, neuroinflammation is induced through the accumulation of Aβ plaques that activate and recruit both microglia and reactive astrocytes. Glial cells are constantly being activated by the excess of Aβ plaques, resulting in the release of inflammatory cytokines [14]. Oxidative stress has been pointed out as playing an important role in mediating inflammation but also as a contributor to its perpetuation, contributing to the pathophysiology of several diseases. In AD, the production of reactive oxygen species (ROS), such as superoxide anion radical (O_2_^•−^) and hydroxyl radical (^•^OH) during the oxidative phosphorylation of ATP, exceeds the antioxidant capacity of the biological environment, causing oxidative damage in biomolecules, such as DNA, lipids, and proteins, also leading to neuronal dysfunction and ultimately death [15].

There are currently no commercially available drugs that can stop the progression of AD, only therapies that can ameliorate the symptoms [16]. The available drugs for the treatment of AD act as cholinesterase inhibitors, NMDAR antagonists, and anti-Aβ antibodies. Donepezil, galantamine, and rivastigmine are cholinesterase inhibitors that were found to improve cognition [9,17,18], especially when used as adjunctive with the NMDAR antagonist memantine [19]. Aβ antibodies are gaining more relevance in the treatment of AD, with lecanemab, aducanumab, and donanemab being recently approved by the Food and Drug Administration (FDA). These antibodies, when used in prodromal AD cases, reduce cognitive decline caused by senile plaque formation and improve memory over the span of six months [20,21]. Despite the beneficial effects of the described anti-AD pharmaceuticals, they tend to cause harsh adverse effects, ranging from general gastrointestinal imbalance, abdominal pain, stomach ulcers, and gastritis to confusion, insomnia, hallucinations, arrhythmia, and convulsions [22,23,24,25]. To reduce inflammatory responses by the brain cells, nonsteroid anti-inflammatory drugs, such as ibuprofen, can be used. These can reduce Aβ formation with minor side effects, such as stomach aches and kidney problems [26]. Unfortunately, due to a lack of human testing and general information, there are currently no sufficient data to support nonsteroid anti-inflammatory drugs use in AD patients [27].

Natural products have been used as traditional medicines for centuries. The scientific community is taking advantage of ancient knowledge by investigating the potential protective effect of natural compounds against AD pathogenesis. For instance, the neolignan 4′-*O*-methylhonokiol, a constituent of the bark of *Magnolia officinalis* Rehder & E. H. Wilson, was found to inhibit astrocyte activation in preselin-2 mutant (PS2) mice brains and other signaling cascades involved in inflammation and oxidative stress, diminishing memory impairment [28]. Caffeic acid, a phenolic compound naturally present in plants, was shown to have neuromodulatory activity via the regulation of LPS-induced neuroinflammation through tumor necrosis factor-α reduction [29]. The protective effect of caffeic acid against AD was assessed in vivo in a high-fat hyperinsulinemic-induced rat model. The administration of 30 mg/kg of caffeic acid for 30 weeks was shown to ameliorate the memory of the animals, as well as affected the major biochemical targets of AD: decreased the expression of phosphorylated (p)-tau protein, attenuated APP expression, and regulated Aβ deposition [30]. Moreover, the compound 2,2′,4′-trihydroxychalcone isolated from *Glycyrrhiza glabra* L. was administrated to a double transgenic mouse model used to study early onset AD. The model APP/PS1 mutant mice express a chimeric mouse/human amyloid precursor protein and a mutant variant of human presenilin 1, both connected to the central nervous system. The compound administered at a 9 mg/kg/day (total treatment 106 days) dosage decreased Aβ production and senile plaque formation, which resulted in memory improvement [31]. Natural compounds with antioxidant properties were shown to prevent free radical formation, thus having a potential neuroprotective effect. The most common antioxidants used in AD treatment are resveratrol, apocynin, and other flavonoids which prevent the inactivation of acetylcholine receptors [32,33,34].

**Figure 1:**
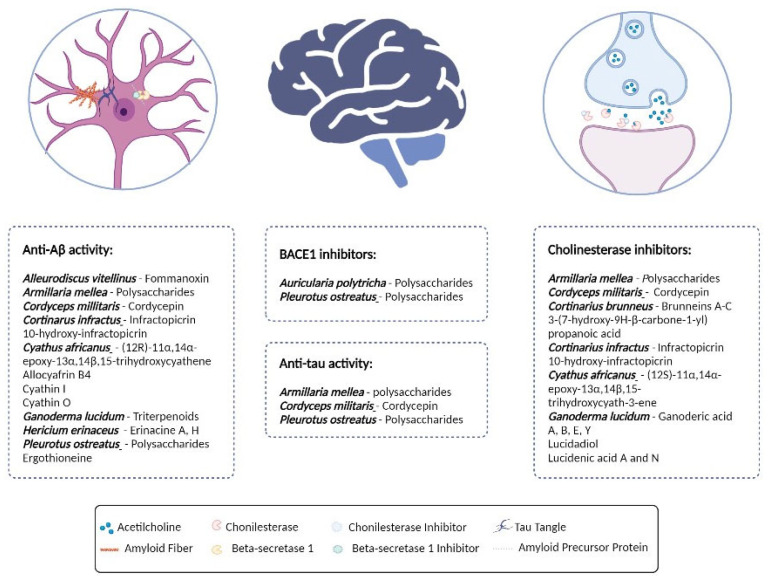
Schematic representation of the main mushrooms bioactive compounds, producer species and AD disease targets. Created with BioRender.com.

A major challenge regarding AD is, in fact, the search for efficient therapies. Presently, most researchers and companies are searching for strategies to discover new targets and new drugs for therapy. The application of natural compounds is among the new features for therapies against AD, as described in recent reviews [25,35,36]. In this field, the potential role of mushrooms for AD treatment or prevention, has been highlighted [37]. Studies using mushroom extracts and compounds have demonstrated neuroprotective, neuromodulatory, and immunomodulatory activities, as well as an anti-inflammatory and antioxidant potential in neurodegenerative diseases [37,38,39]. Well know mushroom species such as *Ganoderma lucidum* (Curtis) P. Karst., *Cordyceps militaris* (L.) Fr. and *Cyathus africanus* H.J. Brodie have been extensively studied with reference to compounds such as ganoderic acid A (**3**), cordycepin (**13**), and cyathins (**5**, **6**), respectively (Figure 1). Although there are several studies in the literature focused on the potential of mushroom extracts and compounds in AD, to the best of our knowledge, there is no publication that specifically compiles information on this subject. Considering the pertinence of the research that links mushrooms to AD, we aim with this review to gather knowledge on the application of mushrooms in AD by compiling the existing data to date.

## 2. Mushrooms in Alzheimer’s Disease 

**Figure 2:**
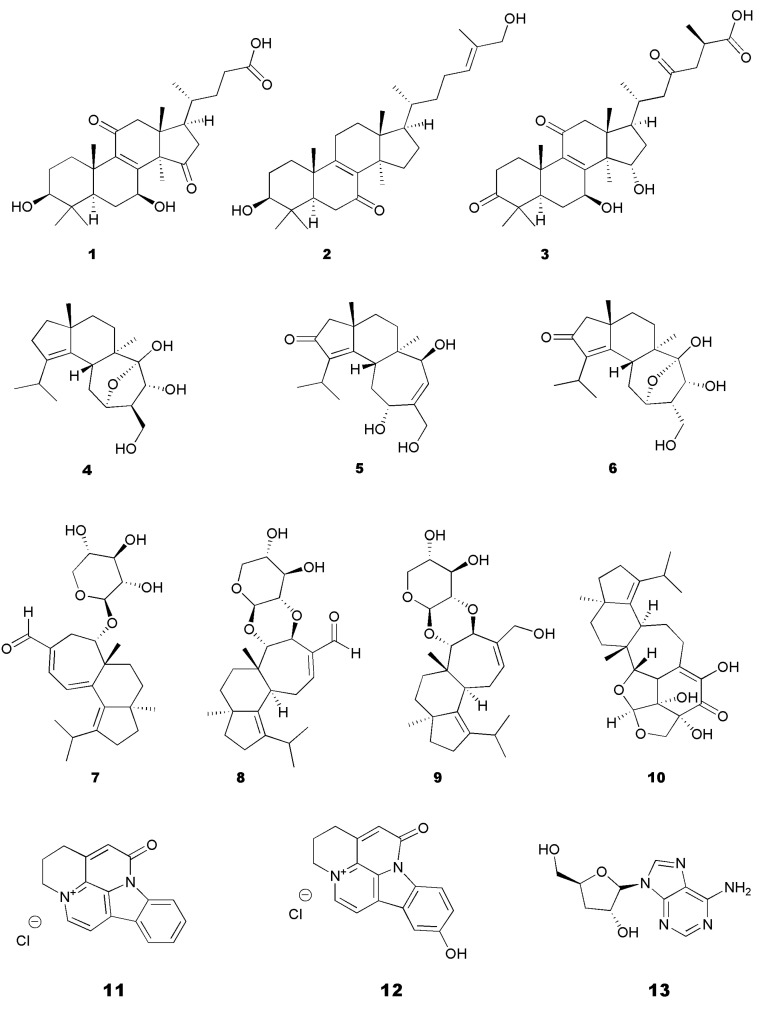
Examples of compounds isolated from mushrooms with potential for AD treatment. These bioactive molecules belong to different classes: lanostane triterpenoids (**1**–**3**), polyoxigenated cyathane diterpenoids (**4**–**9**), sesterterpenoids (**10**), indole alkaloids (**11** and **12**), and purine nucleosides (**13**).

**Figure 3:**
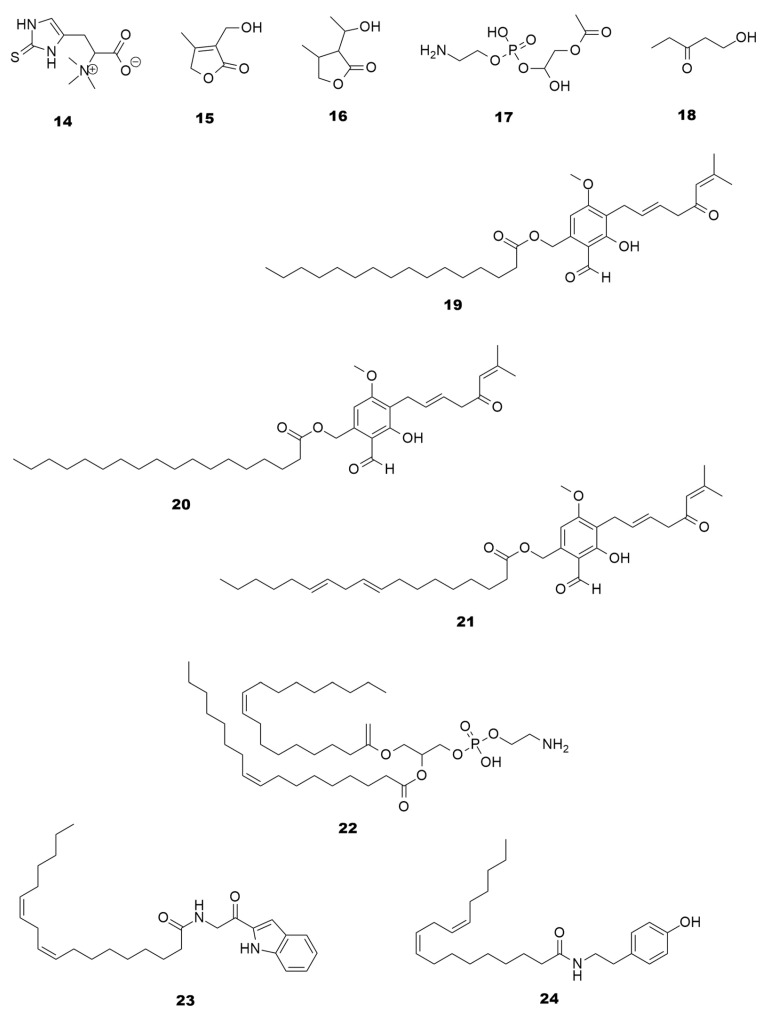
Examples of compounds isolated from mushrooms with potential for AD treatment. These bioactive molecules belong to different classes: amino acids (**14**), lactones (**15** and **16**), benzaldehyde derivates (**19**–**21**), dienamides (**23** and **24**), and ethanolamines (**17**, **18** and **22**).

The number of articles linking mushrooms to AD prevention and/or treatment has increased, especially in the last decade. In fact, it was found that mushroom extracts and compounds were efficient against the main anti-AD-related mechanisms, including the inhibition of BACE1 [10,12], the inhibition of the Aβ aggregation, and the inhibition of AChE and butyrylcholinesterase (BChE) (Figure 1). These can be combined with complementary therapeutic approaches, such as anti-inflammatory or antioxidant therapies, which are known to play an important role in neurodegeneration [13,14,26,40]. Mushroom compounds with anti-AD potential are classified into several classes, including lanostanes, terpenoids, indole alkaloids, purine nucleosides, amino acids, lactones, benzaldehyde derivates, dienamides, and ethanolamines as presented in Figure 2 and Figure 3.

### 2.1. Inhibition of BACE1 and Prevention of Aβ Aggregation and Aβ Cytotoxicity

It has been demonstrated that several mushroom extracts and isolated compounds have anti-BACE1 and anti-Aβ potential (Table 1). Aβ peptide is formed through a cleavage process of APP by the secretases beta (BACE1) and gamma. Aβ peptides tend to interact with each other forming deposits that lead to the formation of amyloid plaques (also referred to as senile plaques), which are considered a hallmark of AD. An excess of Aβ peptide is secreted and accumulates near the synapse. Aβ-dependent neuronal death is thought to be induced due to changes in membrane fluidity and integrity, as these oligomers change the phosphatase and kinase activity, possibly contributing to the hyperphosphorylation of Tau protein. BACE1 has been studied and is considered a promising therapeutic target, as it is involved in Aβ formation [41].

Considering the inhibition of BACE1 and the prevention of Aβ aggregation and Aβ cytotoxicity, species such as *Cordyceps militaris*, *Ganoderma lucidum, Hericium erinaceus* (Bull.) Pers. and *Pleurotus ostreatus* (Jacq.) P. Kumm. are the most representative ones. The mushroom *C. militaris* has been considered one of the oldest sources of bioactive compounds [42]. The first report on the isolation and characterization of *C. militaris* metabolites dates to the 1950s and is related to the purine nucleoside cordycepin (**13**) (Figure 2) [43]. Since then, this molecule has been tested for multiple different purposes, including neurodegenerative diseases, as it exhibits neuroprotective, anti-inflammatory, and immunomodulatory activities [44].

The potential of cordycepin (**13**) for AD has been evaluated in vivo using an Aβ_1–42_-induced mouse model where mice were orally administered 100 and 200 mg/kg/day of cordycepin (**13**) for 15 days [45]. The animals treated with this compound displayed improved cognition, spatial memory, and general behavior (similar results were observed for both concentrations). Rat hippocampal neurons were used as an in vitro model to test cordycepin (**13**) anti-Aβ potential. The results showed that this compound can inhibit Aβ_25–35_ production, as well as Aβ-induced ROS and cytotoxicity, p-tau expression, and AChE activity [46]. 

The medicinal fungus *G. lucidum* has been used in many countries for health promotion [47]. Wang et al. [48] conducted a study to assess the potential effects of diet supplementation with 0.3, 0.6, and 1.8% of *G. lucidum* in senescence-accelerated mice prone 8 (SAMP8). Their results showed no significant differences in body weight, eating habits, or locomotion between the treated and control groups (casein diet). However, a significant difference was observed in the behavioral tests, since the supplemented group showed quicker responses in avoidance tests. A significantly higher SOD, GSH-Px, and GSH-Rd activity, as well as the lower accumulation of Aβ was found in the test groups, showing that *G. lucidum* could be an interesting source of anti-Aβ compounds. Moreover, triterpenoids isolated from *G. lucidum* were discovered to reduce neuronal apoptosis by inhibiting the ROCK-signaling pathway using APP/PS1 mice [49].

Commonly known as Lion’s Mane Mushroom, *H. erinaceus* is frequently used in traditional medicine, due to several health benefits such as anti-inflammatory, antibacterial or anticancer properties [50]. *H. erinaceus* extracts are commercialized as dietary supplements. Several bioactive molecules have been characterized being the most relevant for neurodegeneration erinacine A (**7**) and S (**10**), hericenones (**19**–**21**) (Figure 3), and dilinoleoyl phosphatidylethanolamine (**22**). The protective effects of erinacines A (**7**), C (**9**), and S (**10**) were tested in in vivo using the double transgenic mouse model APP/PS1. The animals were treated with ethanolic mycelium extracts of *H. erinaceus* containing these terpenes at 300 mg/kg/day for 30 days (pathological experimental group) and 70–90 days (behavioral experimental group) [51]. The pathological experimental group showed that the treatment schedule led to an inhibition of Aβ deposition, smaller plaque formation, improvement in the Aβ-degrading enzyme neprilysin, enhancement in nerve growth factor (NGF) production, and a promotion in hippocampal neurogenesis, when compared to control. The mice from the behavioral experimental group showed an improvement in daily life tasks, exhibiting higher nesting scores and nestlet shredding. A similar study conducted in SAMP8 fed with erinacine A (**7**)-enriched mycelium, showed a decrease in Aβ aggregation with an improvement in cognition, memory, and delay in cases of age-related cognitive decline [52]. Tzeng [53] tested the in vivo efficiency of erinacines A (**7**) and S (**10**) in APP/PS1 mice. The results showed that both compounds, at concentrations of 10 and 30 mg/kg/day (100 days) reduced Aβ plaque formation, improved plaque degradation, reduced glial activation, and promoted neurogenesis in the highest concentration. Erinacines A (**7**) displayed the most promising results, with a decrease in Aβ accumulation by inhibiting Aβ production. Behavioral tests also showed an improvement in burrowing and nesting behavior, as well as an increase in spatial learning and memory.

**Table 1:** Summary of mushroom species, extracts, and/or isolated compounds with anti-BACE1 and anti-Aβ aggregation activity tested in different experimental models.

Species	Body Part	Extract/ Compound	In Vitro Assays	In Vivo Assays	Mechanism	Refs.
*Aleurodiscus vitellinus* (Lév.) Pat.	Cultured mycelium	Fomannoxin	Cytotoxicity by MTT; Microfluorimetry of cytosolic Ca^2+^; Immunofluorescence for hippocampal neurons; Thioflavin T binding assay for Aβ aggregation		↓ Aβ-induced cytotoxicity; preservation of synaptic function in hippocampal neurons; ↓ Aβ binding to neurons	[54]
*Amanita caesarea* (Scop.) Pers.	Sporocarp	Aqueous extract	HT22 neuron cells: Apoptosis (annexin V, propidium iodide); MTT; Intracellular ROS; Mitochondrial transmembrane potential (JC-1 staining); Aβ_1–42_ concentration, SOD, ROS levels by ELISA	AlCl_3_, D-galactose induced BALB/c mice: Morris water maze; Rotarod test; Locomotor activity test; Aβ_1–42_ in serum and brain of BALB/c mice measured by ELISA	↓ Aβ_1–42_ in brain; ↑ Aβ_1–42_ in serum	[55]
*Antrodia camphorate* (M. Zang & C.H. Su) Sheng H. Wu, Ryvarden & T.T. Chang	Mycelium and Fruiting body	95% ethanolic extract	PC12 cells: Cytotoxicity by MTT; oxidative stress by DPPH	Aβ_40_ induced Wistar rats: Morris water maze; Immunoblotting (Aβ and BACE1); IHC—Aβ_40_ in hippocampus	↓ Aβ-induced cytotoxicity; ↓ Aβ-induced oxidative stress	[56]
*Armillaria mellea* (Vahl) P. Kumm.	Mycelium	Polysaccharides (precipitated in 70% ethanol)		D-galactose- (D-gal-) induced AD mouse model: Aβ in serum and hippocampus measured by ELISA	↓ Aβ_1–42_ deposition	[57]
*Auricularia polytricha* (Mont.) Sacc.	Whole	HPLC fractions—aqueous, 100% methanol and 10% ethanol	BACE1 inhibition		↓ BACE1;	[46,58]
*Cordyceps militaris* (L.) Fr.		Cordycepin (13)	Hippocampal neurons from rats: Acridine orange staining (Aβ_25–35_); Aβ cytotoxicity by MTT; ROS induction		↓ Aβ_25–35_; ↓ Aβ-induced ROS; ↓ Aβ-induced cytotoxicity	[46]
*Cortinarius infractus* (Pers.) Fr.	Fruiting body	Infractopicrin (11) 10-hydroxy-infractopicrin (12)	HepG2 and SHSY5 cell lines: Cytotoxicity (CytoTox-One assay); AChE inhibition; Thioflavin T binding assay for Aβ aggregation		↓ Aβ_1–40_ aggregation in the absence of AChE	[59]
*Cyathus africanus* H.J. Brodie	Mycelium	(12R)-11α,14α-epoxy-13α,14β,15-trihydroxycyathene Allocyafrin B4 Cyathin I Cyathin O	BV2 microglial cells: Cytotoxicity by MTT; iNOS induction by Aβ_1–42_		↓ Aβ_1–42_ synthesis; ↓ Aβ-induced iNOS	[60]
*Ganoderma lucidum* (Curtis) P. Karst.	Fruiting body	Aqueous	Neurons collected from embryonic Sprague Dawley rats: Caspase-like activity; TUNEL staining; Immunofluorescence staining for synaptophysin and Aβ-induced cytotoxicity		↓ Aβ-induced synaptotoxicity by preserving the synaptic density protein, synaptophysin	[61]
	Whole	Biomass		SAMP8: Open field test Active avoidance test Aging scale	↓ Aβ plaque formation	[48]
		Triterpenoids		APP/PS1 mice: Hematoxylin–eosin staining; IHC; Morris water maze; Exploration test; Burrowing test; TUNEL staining for neuronal apoptosis; Western-blot for Bax, caspase-3, Nrf2, hemeoxidase, NQO1, ROCK-2, GAPDH; SOD; MDA; LDH; Flow cytometry for apoptosis;	↓inhibitory effect of Aβ_25–35_ on neuron proliferation	[49]
*Hericium erinaceus* (Bull.) Pers.	Cultured mycelia	90% Ethanolic extracts containing erinacine A (7), erinacine C (9), and erinacine S (10)		APPswe/PS1ΔE9 double transgenic mouse model (APP/PS1) of AD: Immunoblot; Thioflavin S and AB10 staining for plaque formation and size; ELISA for Aβ levels; Nesting test	↓Aβ deposition; ↓ plaque size; ↑ Aβ-degrading enzymes IDE and neprilysin; ↑NGF	[52]
	Whole	Powdered		AlCl_3_-induced Wistar rats: Morris water maze; Elevated plus maze; Novel object recognition; IHC; Western blot	↓ Aβ_1–42_; ↓ APP	[62]
	Mycelia	Erinacine A (7) Erinacine H		APP/PS1 or SAMP 8 mice: Thioflavin S and AB10 staining for plaque formation and size; ELISA for Aβ; IHC; Immunostaining for Iba-1, AB_10-P_, and glial fibrillary acid protein; Nesting test; Burrowing test; Morris water maze	↓Aβ deposition inhibition; ↓ microglial activation; ↑hippocampal neurogenesis ↑dendritic complexity; ↓ Aβ-induced iNOS	[53]
	Mycelium	Erinacine A (7) enriched mycelium		SAMP8: Avoidance test; iNOS; TBARS; Aβ-aggregation; 8 OHdG for DNA damage	↓ iNOS; ↓ Aβ aggregation	[63]
*Pleurotus ostreatus* (Jacq.) P. Kumm.	Whole	Polysaccharides		Wistar AlCl_3_/D-galactose induced: Morris water maze; Step-down memory test; Western blot for APP, BACE1, Aβ, and, PP2A in hippocampus tissue	↓ Aβ formation; ↓ APP; ↓ BACE1; ↑PP2-A	[64]
		Ergothioneine (14)	HeLa cells: ROS measurement	5XFAD mice Elevated zero maze; Locomotor activity; Rotarod; Novel object recognition; Fear conditioning; IHC; Dynamic PET imaging	↓ Aβ formation	[65]

8-OHdG—8-hydroxy-2′-deoxyguanosine; APP—amyloid precursor protein; BACE1—beta-secretase 1; DPPH—2,2-diphenylpicrylhydrazyl; ELISA—enzyme-linked immunosorbent assay; GAPDH—glyceraldehyde-3-phosphate dehydrogenase; IDE—insulin-degrading enzyme; IHC—immunohistochemistry; iNOS—inducible nitric oxide synthase; LDH—lactate dehydrogenase; MDA—malondialdehyde; MTT-(3-[4,5-dimethylthiazol-2-yl]-2,5 diphenyl tetrazolium bromide; NQO1—NADPH quinone oxiredutase; PP2A—protein phosphatase A; ROCK2—rho-associated protein kinase 2; ROS—reactive oxygen species; SAMP8—senescence-accelerated mouse prone 8; SOD—superoxide dismutase; TBARS—thiobarbituric acid reactive substances; TrkA—tropomyosin receptor kinase A; TUNEL—terminal deoxynucleotidyl transferase dUT; ↑ increase; ↓ decrease.

The species *P. ostreatus* is the second most popular and most cultivated edible mushroom worldwide. Besides being appreciated for its organoleptic properties, *P. ostreatus* has been found to induce different bioactivities, such as antimicrobial, antitumor, antioxidant, and immunomodulatory activities [66]. Zhang et al. [64] tested the potential of *P. ostreatus* polysaccharides to prevent and treat AD. The results of this study showed that administration of 400 mg/kg of polysaccharides extract inhibited BACE1 and Aβ deposition in over 50%, as well as causing a significant inhibition of APP, AChE, and p-tau protein in AlCl_3_- and D-galactose-induced AD Wistar rats, in which aluminum works as a neurotoxic and D-galactose as a senescence stimulating agent, inducing AD-like symptoms. These improvements were studied through behavior tests and revealed that rats treated with *P. ostreatus* polysaccharides had a significant improvement in memory and navigation. Another relevant compound for AD produced by *P. ostreatus* is ergothioneine (**14**), this sulfur-containing amino acid is known for its antioxidant and cytoprotectant properties. Ergothioneine (**14**) was tested in the 5XFAD AD transgenic mouse model, characterized for overexpressing five of the known familial AD mutations related to presenilins 1 and 2 that lead to an increased Aβ deposition, higher plasma, and brain levels of Aβ_40_ and Aβ_42_, along with elevated pro-inflammatory cytokine levels [67]. Results showed ergothioneine (**14**) to have metal chelating activity by capturing heavy or potentially toxic metals in the blood stream and removing them from circulation, as well as ROS scavenging activities and the mitigation of Aβ aggregation, which may explain the improvement of the cognitive deficits observed in the experimental group compared to untreated controls [65].

### 2.2. AChE and BChE Inhibition

One of the most common approaches in AD treatment has been based on repairing cholinergic dysfunction. Cholinergic neurons are involved in several cognitive functions such as memory, attention, learning, and sleep regulation. AD patients have dysfunction in these neurons, mostly in the hippocampus and the neocortex. Damaged cholinergic neurons lead to a decrease in the production of acetylcholine (ACh), a neurotransmitter involved in synapses and general cognition. By lowering the activity of enzymes involved in the degradation of ACh, such as AChE and BChE, the levels of available ACh in the synaptic cleft increase [9,68,69]. When submitted to treatment with ChEIs such as galantamine, a natural compound isolated from *Galanthus nivalis* L., mice showed an improvement in day-to-day tasks, such as burrowing activities, as well as an enhancement in spatial memory and problem-solving activities [70,71].

Several studies with mushroom extracts and compounds were directed to the anti-AD potential by the evaluation of the AChE and BChE inhibition and the activation of ChAT. In Table 2 the main studies found in the literature are presented.

*Ganoderma lucidum* aqueous extracts have been shown to be neuroprotective by displaying interesting antioxidant and anti-inflammatory activities in vivo and in vitro, as well as inhibiting AChE activity [72]. Considering compounds, eighteen different lanostane triterpenes were isolated from *G. lucidum* fruiting bodies. The compounds were tested in vitro and showed AChE inhibition (IC50 ranging between 9.40 and 31.03 µM) [73]. The same study showed that two of the tested compounds, lucidenic acid N (**1**) and lucidadiol (**2**) also inhibited BChE, presenting IC50 below 200 µM [73]. This study showed that *G. lucidium* triterpenes could be potential candidates for anti-AD drugs.

**Table 2:** Summary of mushroom extracts and/or isolated compounds with AChE and BChE inhibitory activity and/or increased ChAT activity tested in different experimental models.

Species	Body Part	Extract/ Compound	In Vitro Assays	In Vivo Assays	Refs.
*Agaricus* *campestris* L.	Fruiting body Whole	Methanol	AChE inhibition; BChE inhibition		[74]
*Amanita* *caesaria* (Scop.) Pers.	Sporocarp	Aqueous	Enzyme inhibition of brain and blood ELISA for AChE, ACh, and ChAT	AlCl_3_, D-galactose BALB/c mice: Morris water maze; Rotarod test; Locomotor activity test	[55]
*Amanita* *crocea* (Quél.) Singer	Fruiting body	Methanolic	AChE inhibition; BChE inhibition		[75,76]
*Armillaria* *mellea* (Vahl) P. Kumm.	Mycelium	Polysaccharides (precipitated in 70% ethanol)		AlCl_3_, D-galactose Balb/c mice: Morris water maze; Fatigue rotarod test; Autonomic activity test; Serum and hypothalamus ACh, AChE, and ChAT; Serum and hypothalamus oxidation status; TUNEL staining; Aβ in serum and hippocampus; IHC	[57]
*Cordyceps* *militaris* (L.) Fr.		Cordycepin (13)	Rat hippocampal neurons: AChE activity induced by Aβ_25–35_		[46]
*Cortinarius brunneus* (Pers.) Fr.	Fruiting body	Brunneins A-C 3-(7-hydroxy-9H-β-carbone-1-yl)propanoic acid	AChE inhibition;		[77]
*Cortinarius* *infractus* (Pers.) Fr.	Fruiting body	Infractopicrin (11) 10-hydroxy-infractopicrin (12)	AChE inhibition; Molecular docking		[59]
*Cyathus* *africanus* H.J. Brodie	Mycelium	(12S)-11α,14α-epoxy-13α,14β,15-trihydroxycyath-3-ene (4) Neocyathin B (5) Neocyathin J (6)	AChE inhibition; Molecular docking		[60,78]
*Cyclocybe cylindracea* (DC.) Vizzini & Angelini	Fruiting body	Methanolic	AChE inhibition; BChE inhibition		[75]
*Ganoderma* *lucidum*	Fruiting body	Aqueous	AChE inhibition		[72]
(Curtis) P. Kumm.	Fruiting body	Ganoderic acid A (3), B, E, Y Ganodermadiol Ganodermanondiol Ganoderiol F Lucidadiol (2) Lucidenic acid A, N (1), Lucidumol B Methyl lucidenate E_2_ Methyl ganoderate A Methyl ganoderate A acetonide n-Butyl ganoderate H n-butyl lucidenate A n-butyl lucidenate N	AChE inhibition		[73]
*Hygrocybe acutoconica* (Clem.) Singer	Fruiting body	Aqueous	AChE inhibition; BChE inhibition		[75]
*Inonotus obliquus* (Ach. ex Pers.) Pilát		Lanostante type triterpenoids	AChE inhibition; BchE inhibition; Molecular docking		[79]
*Morchella* *esculenta* (L.) Pers.	Whole	Polysaccharides	AchE inhibition; BchE inhibition		[80,81]
*Neoboletus erythropus* (Pers.) C. Hahn	Fruiting body	Aqueous	AChE inhibition		[75]
*Phellinus* *pini* (Brot.) A. Ames	Fruiting body	80% methanolic and hot water extracts	AChE inhibition; BChE inhibition	Sprague Dawley rat: Carrageenin-induced hind-paw edema	[82]
*Pleurotus* *ostreatus* (Jacq.) P. Kumm.	Fruiting body	Aqueous	AChE inhibition		[75]
*Russula* *aurea* Pers.	Fruiting body	Aqueous	AChE inhibition		[75]
*Russula* *sanguinea* (Bull.) Fr.	Fruiting body	Aqueous	AChE inhibition in solid and liquid		[75]
*Trametes* *versicolor* (L.) Lloyd	Mycelia and fruiting body	DMSO, ethanol, or sodium phosphate solution extracts	AChE inhibition		[83]
*Trametes* *gibbosa* (Pers.) Fr.	Mycelia and fruiting body	DMSO, ethanol, or sodium phosphate solution extracts	AChE inhibition		[83]
*Trametes* *Hirsute* (Wulfen) Pilát	Mycelia and fruiting body	DMSO, ethanol, or sodium phosphate solution extracts	AChE inhibition		[83]
*Tremella* *fuciformis* Berk.	Fruiting body	Aqueous		Scopolamine-treated Sprague Dawley rat: Morris water maze; IHC for ChAT	[84]
*Tricholoma* *imbricatum* (Fr.) P. Kumm.	Whole	Methanol Hexane Ethyl acetate	AChE inhibition; BChE inhibition		[85]

ACh—acetylcholine; AChE—acetylcholinesterase; BChE—butyrylcholinesterase; ChAT—choline acetyl transferase; DMSO—dimethyl sulfoxide; IHC—immunohistochemistry; ↑ increase; ↓ decrease.

*Amanita caesaria* (Scop.) Pers., commonly known as Caesar’s Mushroom, is a well-appreciated edible mushroom commonly consumed in Turkey and is known to have significant antioxidant and antimicrobial activities, as well as a considerable fatty acid composition [86]. *A. caesaria* aqueous extracts were tested using the immortalized mouse hippocampal cell line HT22 and AlCl_3_, D-gal induced Balb/c mice [55]. Biochemical examinations found that the extracts promoted cell survival and decreased the expression levels of phosphorylated protein kinase B (p-Akt) and the mammalian target of rapamycin (p-mTOR), known to be associated with cellular death, most efficiently at 50 and 100 µg/mL. The in vivo assays consisted of the intragastrical administration of 250, 500, and 1000 mg/kg/day of extract for 28 days. Vertical movement, locomotor activities, and spatial memory were improved when compared to the AD control group (intragastrically administered saline solution). The inhibition of AChE activity was also observed, as well as an increase in ACh and ChAT. The treatment group was also found to have lower levels of Aβ in the brain and reduced levels of ROS and SOD.

The genera *Tricholoma* comes from a diverse mushroom family that is composed of edible and non-edible specimens. Several *Tricholoma* species were found to have antibacterial activity against Gram-negative bacteria [87,88]. In a study developed by Tel et al. [85], the antioxidant and AChE inhibition properties of methanolic, n-hexane, and ethyl-acetate extracts from *T. fracticum* (Britzelm.) Kreisel, *T. terreum* (Schaeff.) P. Kumm. and *T. imbricatum* (Fr.) P. Kumm. were evaluated. Results showed that *T. imbricatum* hexane extract was the most promising, displaying an inhibitory activity of 71.8% for AChE and 52.6% for BChE at 0.2 mg/mL.

The species *Cortinarius infractus* (Pers.) Fr. is an inedible mushroom known to have a bitter taste due to its rich composition of infractopicrins (**11**) [89]. Compounds **11** and 10-hydroxy-infractopicrin (**12**) showed a significant reduction in AChE activities (IC50 9.72 and 12.7 µM, respectively). The two indole alkaloids also displayed inhibitory activity against Aβ_1–40_ formation at 25 µM [59]. 

### 2.3. Tau Protein Expression and Aggregation

Tau is a protein involved in microtubule formation and function. This protein plays a significant role in cytoplasmatic transport and allows synaptic function as well as the maintenance of their structure, regulating neuronal signaling. The hyperphosphorylation and abnormal cleavage of Tau leads to deficient microtubule activity due to depolymerization consequently causing a loss of neuronal morphology as well as impairments in axon and dendrite formation [6,11]. NFTs are then formed, causing a neuronal malfunction due to impaired synaptic function and neurotoxicity. The level of Tau hyperphosphorylation seems to correlate with the severity of the AD scenario in a direct way [6,11]. In Table 3, the most relevant studies linking mushrooms to protein Tau are compiled followed by a description of the most representative species.

*Armillaria mellea* (Vahl) P. Kumm. is an edible medicinal mushroom used in traditional medicine in East Asia [90]. Polysaccharides isolated from *A. mellea* have been reported as having interesting bioactivities, such as antioxidant activity due to radical scavenging potential. A study conducted by An et al. [57] intended to better understand the mechanisms behind *A. mellea* polysaccharides protection by using an in vitro and an in vivo approach. In vitro, results showed that these molecules improved cell viability in mouse hippocampal neuronal cell line HT22 by inhibiting caspase-3 activity, restoring mitochondrial membrane permeability, and reducing ROS accumulation. As for the in vivo approach, D-galactose-induced Balb/c mice were treated with 25 and 100 mg/kg/day for four weeks. The supplementation with *A. mellea* polysaccharides regulated AChE, ACh, and ChAT and Aβ concentration in mice serum and hypothalamus (Table 1 and Table 2) as well as a significant reduction in p-tau aggregations in the hippocampus of treated mice at 25 and 100 mg/kg/day. 

**Table 3:** Summary of mushroom extracts and/or compounds with anti-Tau activity tested in different experimental models.

Species	Body Part	Extract/ Compound	In Vitro Assays	In Vivo Assays	Mechanism	Refs.
*Antrodia camphorata* (M. Zang & C.H. Su) Sheng H. Wu, Ryvarden & T.T. Chang	Mycelium Fruiting body	Aqueous Ethanolic		Aβ-induced Wistar rats: Immunoblotting; Morris water maze;	↓ p-tau expression	[56]
*Armillaria* *mellea* (Vahl) P. Kumm.		Polysaccharides		AlCl_3_, D-galactose-induced Balb/c mice: Morris water maze; Fatigue rotarod test; Autonomic activity test; TUNEL staining	↓ p-tau aggregation; Antioxidant activity	[57]
*Cordyceps* *millitaris* (L.) Fr.		Cordycepin (**13**)	Rat hippocampal neurons: Western blotting for p-tau		↓ Aβ_25–35_-induced p-tau expression	[46]
*Hericium* *erinaceus* (Bull.) Pers.	Whole	Powdered		AlCl_3_-induced Wistar rats: Morris water maze; Elevated plus maze; Novel object recognition; IHC; Western blot	↓ p-tau	[62]
*Pleurotus* *Ostreatus* (Jacq.) P. Kumm.	Whole	Polysaccharides		Wistar AlCl_3_/D-galactose-induced rats: Morris water maze; Step-down memory test; Serum, hippocampus, and liver levels of SOD, GSH-Px, CAT, and MDA; Western blot for p-tau in hippocampus tissue	↓ p-tau	[64]

CAT—catalase; GSH-Px—glutathione peroxidase; IHC—immunohistochemistry; iNOS—induced nitric oxide synthase; MDA—malondialdehyde; p-tau—phosphorylated tau; SOD—superoxide dismutase; TUNEL—terminal deoxynucleotidyl transferase dUTP; ↑ increase; ↓ decrease.

As already described, cordycepin (**13**) isolated from *C. militaris* was found to inhibit Aβ-induced apoptosis in hippocampal cultivated neurons and to inhibit AChE activity. At 10 µM, the compound also decreased p-tau expression through an adenosine A1 receptor-dependent mechanism.

### 2.4. Other Activities for General Neuronal Protection

Neuroprotection is generally defined as a physiological or induced measure to preserve neurons. This can be achieved through a therapeutic approach resulting in the salvage, recovery, or even regeneration of the central nervous system cells, structure, and function. Classical examples of neuroprotectors are molecules that intervene in neurotransmitter receptors pathways via agonist/antagonist interaction. These have been explored for their neuroprotective activity as well as their disease-modifying potential. Table 4 summarizes the most relevant mushroom extracts and isolated compounds with neuroprotective potential other than the effects described in the classical AD hypothesis and antioxidative and anti-inflammatory potential.

*Grifola frondosa* (Dicks.) Gray is an edible mushroom found in America, Europe, and Asia with nutritional and medicinal properties due to its rich composition in bioactive molecules, namely polysaccharides, β-glucans, and heteroglycans [91]. Similarly to members of the *Ganoderma* genus, *G. frondosa* is known for its medicinal properties and is used to treat diverse ailments by indigenous Malaysian tribes [92]. An in vivo study by Ling-Sing Seow et al. [93] explored the neuroprotective activities of *G. frondosa*, *G. lucidum,* and *G. neo-japonicum* Imazeki aqueous extracts by testing different concentrations until 2500 ganoderma neo µg/mL in the rat pheochromocytoma cell line PC12, known to have similar behaviors as mature neuronal cells [94]. The results revealed that all extracts stimulated NGF production. While *G. frondosa* and *G. lucidum* extracts stimulated NGF production at 12.60 and 12.07% at 75 µg/mL, *G. neo-japonicum* stimulated NGF production at 14.0% at 50 µg/mL. Lysophosphatidylethanolamine (**17**), a phospholipid isolated from *G. frondosa*, was shown to not only stimulate NGF but also enhance neuronal outgrowth and neurofilament M expression while stimulating cell proliferation and survival through the activation of the Ras/MAPK signaling pathway [95].

The lanostane triterpenoid ganodenic acid A (**3**) isolated from *G. lucidum* has been shown to inhibit cell apoptosis in brain and liver tissues through the mTOR pathway, while also promoting autophagy, when administered in concentrations of 10 mg/kg/day, when compared to control (treated with normal commercial feed) [96]. 

Other biologically interesting metabolites produced by *H. erinaceus* besides erinacines A-C (**7**–**9**) include hericenones and dioleoylphosphatidylethanoleamine (**22**). Hericenones (**19**–**21**) are a vast family of benzaldehyde derivates while compound **22** belongs to the phosphatidylethanolamine family. Both are known to have neuroprotective properties [97,98,99]. Hericenones C-E (**19**–**21**) have been tested in PC12 cells showing the ability to stimulate NGF production through TrkA (tropomyosin kinase A) and MEK (mitogen-activated kinase), as well as neuronal outgrowth mediated by Pi3K/Akt signaling pathway [98,99]. Dilinoleoyl phosphatidylethanolamine (**22**) extracted from *H. erinaceus* was tested in Neuro2a cells and showed neuroprotective potential by diminishing endoplasmic reticulum stress-induced death [97]. *Termitomyces titanicus* Pegler & Piarce-derived compounds, such as 3-(hydroxymethyl)-4-methylfuran-2(5H)-one (**15**), (3R,4S,1R)-3-(1′-hydroxy-ethyl)-4-methyldihydrofuran-2(3H)-one (**16**), and 1-hydroxy-3-pentanone (**18**), are found in *Mycoleptonoides aitichisonii* Karasaki [100] and the fatty acid amides termitomycamides B (**23**) and E (**24**) also displayed neuroprotective activities [101]. 

**Table 4:** Summary of mushroom extracts and isolated compounds with neuroprotective activity tested in different experimental models.

Species	Body Part	Extract/ Compound	In Vitro Assays	In Vivo Assays	Mechanism	Refs.
*Armillaria* *mellea* (Vahl) P. Kumm.	Mycelium	Polysaccharides (precipitated in 70% ethanol)	HT22 apoptotic cells: ROS inhibition; MMP depolarization	AlCl_3_, D-galactose Balb/c mice: Morris water maze; Fatigue rotarod test; Autonomic activity test; IHC	↑ Cell viability; ↑Behavior	[57]
*Cordyceps* *millitaris* (L.) Fr.		Cordycepin (**13**)		Aβ_1–42_ induced ICR mice: T maze test; Novel object recognition test; Morris water maze; NO scavenging activity	↑Spatial memory ↑Behavior; ↑Memory	[45]
*Coriolus* *versicolor* (L.) Quél.	Whole	Biomass		C57BL/6 WT mice: Immunofluorescence; Microscopy for dendritic morphology assessment and β-catenin quantification; Mouse body conditions	↑ Arborization of newly generated neurons; ↑ β-catenin	[102]
*Dictyophora indusitata* (Vent.) Desv.		Dictyophorine A Dictyophorine B	Quiescent rat astroglial cells: NGF production		↑NGF	[103]
*Ganoderma* *lucidum* (Curtis) P. Kumm.	Fruiting body	Aqueous	Neurons collected from embryonic Sprague Dawley rats: Aβ-induced cytotoxicity; Caspase-like activity		↓ JNK phosphorylation; ↓ Caspase-3-like activity	[61]
	Basidiocarp	Aqueous	PC-12 cells: Cell viability by MTT; Neurite outgrowth stimulation; Immunofluorescence		↑NGF	[93]
		Ganodenic acid A (**3**)		C57 BL/6 APP/PS1 mice: IHC; Western blotting; Transcriptome sequencing; Metabolic analysis	↑p-mTOR; ↓ apoptosis; ↑sphingolipid metabolism; ↑ iron function; ↑autophagy; Regulation of lipid metabolism;	[96]
*Ganoderma neo-japonicum* Imazeki	Basidiocarp	Aqueous	PC-12 cells: Cell viability by MTT Neurite outgrowth stimulation Immunofluorescence		↑NGF	[93]
*Grifola* *frondosa* (Dicks.) Gray		Lysophosphatidylethanolamine (**17**)	PC12 cells: MAPK activation; Protein phosphorylation; Signal transduction inhibition; Apoptotic DNA fragmentation		↑ neurite outgrowth- GLPE; ↑ neurofilament M expression; ↑Ras/MAPK; ↑NGF	[95]
	Basidiocarp	Aqueous	PC-12 cells: Cell viability by MTT; Neurite outgrowth stimulation; Immunofluorescence		↑NGF	[93]
*Hericium* *erinaceus* (Bull.) Pers.	Basidiocarp	Hot water and 80% ethanolic extract	NG108-15 (neuroblastoma glial cell hybrid) and MRC-5 (human lung fibroblast) cell lines: Cell viability by MTT, trypan blue, tunnel assay; Neurite outgrowth stimulation assay; ELISA for NGF		↑NGF	[104]
		Hericenone C (**19**), hericenone D (**20**), hericenone E (**21**)	PC12 cells: ELISA for NGF; Enzymatic inhibition for MAPK, PI3K, TrkA; Phospho-ERK levels; Phospho-Akt levels		↑NGF (mediated by TrkA, MEK); ↑ Neurite outgrowth (mediated by Pi3K/Akt)	[99]
	Mycelia	Erinacine A–C (**7**–**9**)	Mouse astroglial cells: Medium NGF		↑NGF	[105]
		Erinacine A (**7**)		Wistar rats: Catecholamine levels (tissue); Indoleamine levels (tissue); ELISA for NGF;	↑NGF; ↑Catecholamine	[106]
		dilinoleoyl phosphatidylethanolamine (**22**)	Neuro2a: Cell viability by MTT; PI staining; Caspase-12 activation; Protein kinase C activation		↓ Endoplasmic reticulum stress-induced death; ↑PKC	[97]
*Mycoleptodonoides aitchisonii* Karasaki	Whole	Aqueous; Powder	Rat astrocytes: NGF	Newborn Wistar rats: Morris water maze; Monoamine concentration; Amino acid concentration; ELISA for NGF	↑NGF; ↑L-serine; ↑spatial memory	[107]
		Terpenoids: 3-(hydroxymethyl)-4-methylfuran-2(5H)-one (**15**) (3R,4S,10R)-3-(1′-hydroxy-ethyl)-4-methyldihydrofuran-2(3H)-one (**16**) 1-hydroxy-3-pentanone (**18**)	Neuro2a cells: Cell viability by MTT		↓ Endoplasmic reticulum stress-induced death; ↑NGF	[100]
*Paxillus* *panuidodes* (Fr.) Fr.		p-terphenyl leucomentins 2–6	Mouse cortical cells: Cell viability by MTT; NMDA cytotoxicity; Glutamate cytotoxicity; Iron chelation—DNA damage		↓ NMDA cytotoxicity; ↓ Glutamate cytotoxicity; ↓DNA damage; ↓ Fenton reactions	[108]
*Sarcodon* *cyrneus* Maast Geest.		Cyrneine A Cyrneine B	PC12 and 1321N astrocytoma cells Medium NGF levels		↑ Neurite growth	[109,110]
*Sarcodon scabrosus* (Fr.) P. Karst.		Scabronine A	1321N astrocytoma cells: Medium NGF levels; RT-PCR		↑ NFG synthesis	[111]
		Scabronine B–F	Rat astroglial cells: Medium NGF levels		↑ NFG synthesis	[101]
*Termitomyces albuminosus* (Berk.) R. Heim		Termitomycesphins A–D	PC-12 cells: Neurite growth		↑ Neurite growth	[112]
		Termitomycesphin E–H	PC12 cells Morphology monitoring—phase contrast microscopy		↑ Neurogenesis	[113]
*Termitomyces titanicus* Pegler & Piearce		Termitomycamides B (**23**) and E (**24**)	Neuro2a cells: Tunicamycin-induced stress test		↓ Endoplasmic reticulum stress-induced death	[114]
*Tremella* *fuciformis* Berk.	Fruiting body	Aqueous	PC12h cells: Morphology evaluation by imaging	Scopolamine-treated Sprague Dawley rat: Morris water maze	↑ neuritogenesis; ↑ NGF; ↑ FGF	[84]

ELISA—enzyme-linked immunosorbent assay; ERK—extracellular-signal-regulated kinase; FGF—fibroblast growth factor; GLPE—thiosulfate transferase; IHC—immunohistochemistry; JNK—jun-amino-terminal kinase; MAPK—mitogen-activated protein kinase; MEK—mitogen-activated kinase; MMP—mitochondrial membrane permeability; MTT- (3-[4,5-dimethylthiazol-2-yl]-2,5 diphenyl tetrazolium bromide; NGF—nerve growth factor; NMDAR—N-methyl-D-aspartate receptor; Nrf2—nuclear factor erythroid 2-related factor 2; p-mTOR—phosphorylated mammalian target of rapamycin; pi3K—phosphoinositide 3-kinase; PKC—protein kinase C; RT-PCR—real time polymerase chain reaction; TrkA—tropomyosin receptor kinase A; ↑ increase; ↓ decrease.

As previously described, inflammatory and oxidative processes have been revealed to be highly deleterious in neurodegeneration scenarios. It is known that mushrooms are composed of many compounds with antioxidant and anti-inflammatory molecules, such as polysaccharides, β-glucans, and phenolic/aromatic compounds. A sizable number of mushroom-isolated compounds display anti-inflammatory and antioxidant activity; therefore, most extracts and compounds described in the previous tables display such activities, thus enhancing the potential of mushroom-derived compounds as possible anti-AD treatment or adjuvants [115]. A recent review by Abitbol et al. [37] efficiently covers the relationship between neuro-inflammation and the potential use of mushroom-derived compounds in AD, for this reason, the topic was not discussed in the present review.

A multi-target therapeutic approach is believed to be more effective due to the multifactorial nature of AD and the fact that some of the hallmark mechanisms are closely related to the point of complementing the pathways involved in other mechanisms [116]. As presented in Table 1, Table 2, Table 3 and Table 4, several mushroom extracts and isolated compounds display one or more anti-AD activities. There are a few examples of extracts displaying more than one activity, including the *A. camphorata* mycelium and fruiting body 95% ethanolic extract, which reduced Aβ-induced cytotoxicity, oxidative stress, and p-tau expression [56]. *G. lucidum* fruiting body aqueous extract, was shown to inhibit Aβ-induced cytotoxicity, AChE, and caspase 3-like activity, thereby enhancing cell viability as well as NGF expression [61]. *Tremella fuciformis* Berk. fruiting body aqueous extract inhibited cholinesterase activity while improving neuritogenesis by stimulating NGF and FGF expression [84]. The most notorious anti-AD mushroom-derived compounds are cordycepin (**13**) isolated from *C. militaris,* neocyathins B (**5**) and J (**6**) from *C. africanus*, and erinacine A (**7**) from *H. erinaceus*. Cordycepin (**13**) caused an inhibition in Aβ_25–35_ production, while reducing Aβ-induced ROS, toxicity, AChE, and tau, resulting in an improvement in spatial memory when administered to mice [46]. Compounds **5** and **6** significantly inhibited Aβ_1–42_ production, as well as Aβ-induced iNOS and AChE activity [60]. Erinacine A (**7**) inhibited Aβ deposition, Aβ-induced iNOS, and microglial activation while stimulating neurogenesis through enhanced NGF production [53]. Isolated polysaccharides have also shown interesting combined bioactivities, such as *P. ostreatus* polysaccharides that displayed neuroprotector activity, inhibiting Aβ formation, APP expression, BACE1 activity, and p-tau deposition [64]. The most interesting results have been found in extracts compared to isolated compounds and compound groups, which can possibly be explained by existing synergistic interactions between compounds in the more diverse extract components. 

The results mentioned above highlight the known anti-AD potential exhibited by some extracts and isolated compounds from mushrooms. Although the presented results appear to be interesting, there is still a lack of knowledge about the mechanisms underlying anti-AD protection, which enhances the need for additional research and new isolated molecules that can be applied to anti-AD therapeutics.

### 2.5. Medicinal Mushrooms in AD Clinical Studies 

Mushroom extracts and derivates have been widely accepted as supplements and are thought to improve general health in different ways, such as diminishing chemotherapy side effects; helping with insomnia, anemia, or neurasthenia; and even improving memory [117,118]. 

Despite the evidence of the anti-AD potential of mushroom extracts and isolated compounds obtained in pre-clinical trials, there is still a lack of published clinical trials. 

Some exceptions are clinical trials where patients were treated with *Hericium erinaceus*. In a double-blind, parallel-group, placebo-control trial that included 30 patients suffering from mild cognitive impairment, 1000 mg of 96% pure mushroom powder was administered three times a day for 16 weeks [119]. Follow-up was performed for 4 weeks after the last dosage and cognitive function was evaluated using a cognitive function scale based on the revised Hasegawa dementia scale. Results showed that, compared to the placebo group, the experimental group exhibited an improvement in cognitive function. Although, at the end of the follow-up, there was a significant decline in results, no major adverse effects were reported with only one participant leaving the study with stomach pain complaints. Additionally, in a more recent study conducted by Li et al. [120] aiming to assess *H. erinaceus* mycelium enriched with erinacine A (**7**) potential against early AD, a randomized, double-blind, placebo-controlled pilot study was conducted for 49 weeks. In this trial, patients were administered three capsules daily containing 350 mg of *H. erinaceus* mycelium and 5 mg/g of compound **7**. When compared to the placebo control, the results pointed to an improvement in the instrumental activities of the daily living score as well as Mini-Mental State Examination score, and contrast sensitivity. Concerning AD-specific markers, the experimental group was found to have reduced apolipoprotein APOE4 expression as well as lower concentrations of Aβ_1–40_ and higher concentrations of brain-derived neurotrophic factor. Four patients left the trial after suffering from abdominal discomfort, nausea, and skin rash. Although not directly connected to AD, during a study concerning the effects of compound **7** enriched *H. erinaceus* on hearing impairment, Chan et al. [121] found that the administration of 2000 mg/day combined with 10 mg of compound **7** caused a significant increase in serum NGF and brain-derived neurotrophic factor. All of these studies indicate that this medicinal mushroom could be useful as a possible treatment or adjuvant for anti-AD treatment.

## 3. Conclusions

Given the incidence, severity, and complex nature of Alzheimer’s disease, all efforts made towards finding a cure are critical. Mushrooms are a source of pharmacologically diverse and interesting molecules, produced by different species and body parts. By linking these two subjects, the present review provided an overview of the potential anti-AD properties of mushrooms. Throughout the literature, the link between mushrooms and the disease was obvious and the number of compounds and number of extracts with activity falling within the AD main hypotheses were surprising. 

AD is a complex disease with multiple factors involved, which makes it challenging to determine the most crucial mechanisms. However, multi-target inhibitors appear to be the most promising option, as observed in some of the mushroom-derived compounds. These compounds have the potential to be used in anti-AD therapy or as adjuvants in existing treatments. However, although mushroom extracts and derived molecules have been extensively investigated both in vivo and in vitro, currently few clinical trials have been conducted. 

Global mushroom production has increased in recent decades and is expected to further increase in the future. As mushrooms are widely consumed all over the world, and their consumption is well accepted when introduced into the market, the knowledge that they can play a positive role in the process of neurodegeneration and cognitive health is valuable and reinforces the necessity of research in this area.

## Data Availability

Not applicable.
